# Influence of Rurality on Oral Cancer Trends among Organisation for Economic Co-Operation and Development (OECD) Member Countries—A Scoping Review

**DOI:** 10.3390/cancers16172957

**Published:** 2024-08-24

**Authors:** Poornima Ramamurthy, Dileep Sharma, Alan Clough, Peter Thomson

**Affiliations:** 1 College of Medicine and Dentistry, James Cook University, Cairns 4870, Australia; 2 Eleanor Duncan Aboriginal Services, Mardi 2259, Australia; 3 School of Health Sciences, The University of Newcastle, Ourimbah 2258, Australia; 4 College of Public Health, Medical and Veterinary Sciences, James Cook University, Cairns 4870, Australia; alan.clough@jcu.edu.au; 5 School of Medicine and Dentistry, Griffith University, Southport 4215, Australia; p.thomson@griffith.edu.au

**Keywords:** oral cancer, incidence, prevalence, risk factors, rural and remote population, OECD member countries

## Abstract

**Simple Summary:**

Oral cancer affects the mouth and throat areas. It is a major cause of death for older people in developed countries. This review looked at how living in rural areas influences oral cancer trends in these countries. The studies from these countries showed increasing rates of oral cancer in rural areas of the US, Australia, Canada, and Europe. Older people are more affected by these cancers than younger groups. The main risk factors are tobacco use, alcohol consumption, and HPV infections. People in rural areas often do not know much about HPV-related cancers. They also tend to use more tobacco and alcohol than city dwellers. Even in developed countries, living in rural areas can lead to shorter lifespans for oral cancer patients. This is mainly because it is harder for them to access specialized cancer treatment centres and advanced medical care. In summary, where people live can significantly impact their chances of surviving oral cancer, even in wealthy nations.

**Abstract:**

Oral cancer is the general term used to describe cancers of the oral cavity and oropharyngeal region. These cancers are one of the leading causes of death in elderly residents within the Organisation for Economic Co-operation and Development (OECD) member countries in the 21st century. This scoping review was carried out to assess the influence of rurality on oral cancer trends and patterns among OECD member countries. Four online databases (Medline, PubMed, Scopus, and CINAHL) were searched for studies that reported on oral cancer trends in rural and remote areas in OECD member countries. A total of 1143 articles were obtained initially; among them, 995 papers were screened to include 18 articles for this scoping review. Studies have reported increasing incidence and prevalence in the United States, Australia, Canada, and European countries wherein risk factors such as tobacco, alcohol, and human papilloma virus (HPV) infections were associated with oral and oropharyngeal cancers. Awareness among people living in rural areas about HPV-related cancers was very low, while rates of tobacco and alcohol abuse were noted to be rising more rapidly than among their urban counterparts. Furthermore, the ageing population was most affected compared to the younger age groups of people with oral and oropharyngeal cancer that are prevalent in these regions. Overall, despite living in developed countries, rurality was noted to be a significant factor in the lower life expectancy of oral cancer patients, mainly due to the limited accessibility to tertiary cancer care centres and advanced medical care.

## 1. Introduction

Oral cancer is one of the leading causes of death among head and neck cancers worldwide [1]. Malignancies can occur in any tissue in the head and neck region, but most tumours in this region develop from the mucosa lining the upper aerodigestive tract and are more likely to be classified as squamous cell carcinoma (SCC) [2]. The recent literature suggests an increasing trend in the development of oral cancer among rural populations around the globe [3,4]. Notably, residents of rural areas experience below-average life expectancy compared to urban dwellers attributable to various sociodemographic and geographic factors, such as age, gender, rurality, low health awareness, and educational and employment levels, along with limited access to healthcare services [5,6,7,8,9,10,11].

The Organisation for Economic Co-operation and Development (OECD) member countries and key partners represent 80% of world trade and investment [12]. In regard to rural and remote populations in OECD member countries, the available evidence indicates that several factors contribute to an increasing trend of oral and oropharyngeal cancers [13]. In case of OECD member countries, rural populations are at a high risk of head and neck carcinoma (HNC) and lower life expectancy in comparison to their urban counterparts, either due to their remoteness from the tertiary healthcare centres or the increased burden from family life, work, financial, and educational factors which influence the overall quality of life [13].

Since the beginning of the 21st century, multiple studies have been conducted globally to assess the trends of oral and oropharyngeal cancers in rural and remote communities, including a recent systemic review focusing on rural United States [14]. In the United States, cancer is the second leading cause of death with an estimated 370,300 people diagnosed with some form of head and neck cancer [15]. Multiple studies conducted in the USA suggested that increased oral cancer incidence was associated with rurality and poverty in men of African-American ethnicity [5,16,17,18,19,20,21]. In another study, lifetime HPV risk in young adults was noted to be higher in males (91.3%) than females (84.6%) [22]. Other factors that play an important role include the following: reduced access to healthcare facilities; unemployment; sedentary lifestyle; and lower community cohesion [23]. Interestingly, oral cavity cancers were common in older males, particularly in African-American men, but oropharyngeal cancers were more commonly noted in younger Caucasian males [24]. The association between HPV and oropharyngeal cancer showed significant variation with increased knowledge about HPV in Caucasians compared to African Americans [17,18]. However, tobacco and alcohol abuse were the dominant risk factors for SCC among both the races [18]. A 225% increase in HPV-associated oropharyngeal cancer between 2010 and 2016 was also noted [25]. Studies conducted in Florida confirmed that rural residents lacked knowledge of oral and oropharyngeal cancer compared to their urban counterparts, suggesting a need to educate the population in rural areas about the risk factors and symptoms of oral cancer [26].

In Europe, oral and pharyngeal cancers are the seventh leading type of cancer. In Germany alone, there are more than 10,000 new cases diagnosed annually [12]. A total of 3127 new cases of oral and pharyngeal cancer were recorded between 2000 and 2006 within the state of Schleswig–Holstein, Germany, with more than 80% of all cases diagnosed as SCC [12]. Another German study conducted in rural communities with a high prevalence of HPV infection showed its significant influence and resulted in highly variable numbers of incidence rates of SCC [27]. Overall, studies conducted in the USA, Canada, Germany, Spain, French territories, and in Australia all reported an increased rate of incidence in rural communities compared to their urban counterparts [2,12,13,15,18,23,25,26,27,28,29,30,31,32,33,34,35,36,37,38,39,40,41,42].

Australian studies point towards an increasing number of oral cancer cases diagnosed in the advanced stages of the disease with reasons for a delay in diagnosis including regional isolation, a lack of knowledge about the risk factors and symptoms, tobacco and alcohol abuse, and HPV-related oropharyngeal squamous cell carcinoma [38,40,41]. A high prevalence of tobacco and alcohol consumption along with betel (areca) nut consumption was noted among Chinese, South Asian, and Taiwanese immigrants living in regional Canada with low socioeconomic profiles, which contributed significantly to the increased incidence of oral cavity cancer among older age group men [23].

While there are multiple studies that have been published from OECD countries, there is no clear consensus on the influence of rurality on oral and oropharyngeal cancer trends in most OECD member countries. Hence, this scoping review focuses on the oral cancer trends in the rural and remote communities of the OECD member countries. The objective of this review is to identify the gap in research on oral cancer trends and the influence of rurality on these trends.

## 2. Methods

The framework for this scoping review, described below, was adapted from the methodology described by Arksey and O’Malley and Sucharew et al. to ensure comprehensive and reproducible, structured literature searches were completed for relevant information, with minimal bias [2,43,44]. All the steps for conducting a scoping review as outlined by Arksey and O’Malley were followed except the optional consultation, since this was not feasible in our context [43,44]. The scoping review and the search strategy were driven by the following research question.

How does rurality influence oral cancer trends in OECD member countries?

### 2.1. Inclusion Criteria

Retrospective and observational studies which assessed the oral cancer trends, such as risk factors, incidence, and prevalence of oral cancer, with no age or gender restrictions, published in the English language, and studies carried out in rural populations of OECD member countries from 2000 onwards were included in this scoping review, to assess the OSCC trends in the 21st century.

### 2.2. Exclusion Criteria

Any type of studies carried out in countries other than OECD member countries, focusing on cancer in regions other than the oral cavity and oropharynx, systematic, scoping or narrative literature reviews, animal studies, opinion papers, conference papers, case reports, case series, and articles published in non-English languages were excluded. Additionally, studies that included interventions or solely focusing on the urban population were also excluded from this review.

### 2.3. Search Strategy

After discussion with a research librarian, the formulation of the search strategy was finalised to identify suitable keywords for electronic database searches. The Medline (Ovid), PubMed, Scopus, and CINAHL databases were searched using the relevant keywords. Supplementary Table S1 consists of the search strategy and the search strings used to retrieve studies included in this review.

### 2.4. Study Selection

Two of the reviewers independently screened all the titles and abstracts and excluded the studies which did not meet the inclusion criteria. Disagreements between the two reviewers were resolved by the third reviewer. The Endnote (v20.3 Clarivate, Philadelphia, PA, USA) bibliographic software was used to import, screen, and manage the references.

## 3. Results

**Figure 1:**
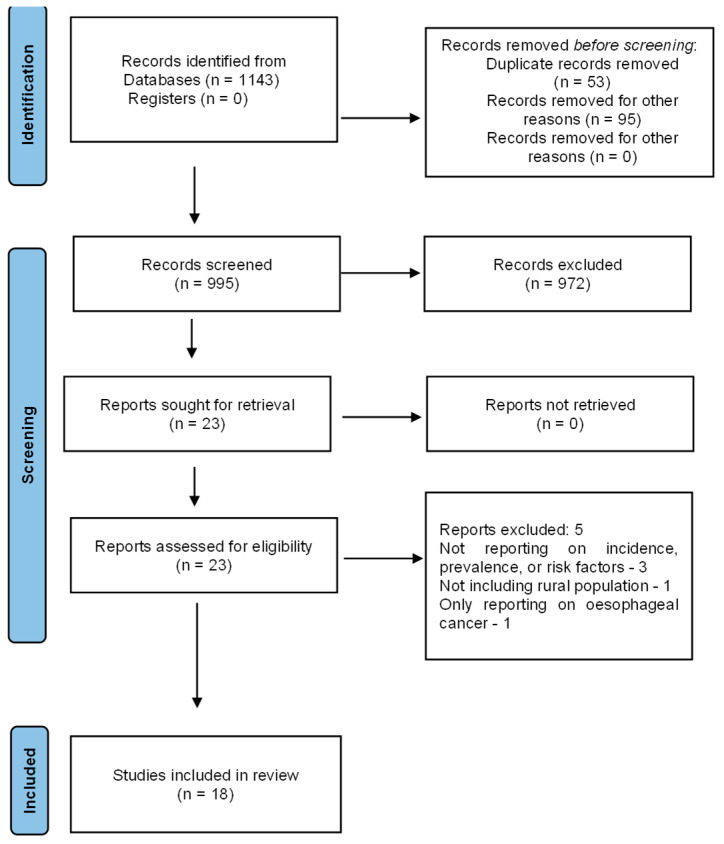
PRISMA diagram of the literature search strategy for scoping review.

The initial search from all the four databases yielded a total of 1143 articles. After removing the duplicates, 995 articles were screened for inclusion by title and abstract review. The reviewers agreed to consider 22 articles for full text review and final inclusion. Based on the predetermined inclusion and exclusion criteria, 18 papers were included for this scoping review. Additionally, reference lists of these papers were searched to ensure that all the relevant studies were included in this review. The Preferred Reporting Items for Systematic review and Meta-analysis (PRISMA) flow chart illustrates the literature search and selection process followed in this scoping review (Figure 1). The main findings and basic characteristics of the articles included in this scoping review are presented in Table 1.

Studies conducted on cancer databases from Canada, USA, France, Germany, and Australia have reported incidence of oral and oropharyngeal cancers and the factors contributing to the increase in incidence and mortality, such as, socioeconomic deprivation, suburbanisation, aged population, rurality, and tobacco and alcohol abuse, along with HPV-related oral cancer. Eleven of the studies focused on the incidence rates including data on some or all the above parameters [14,16,17,20,23,34,35,40,41,45,46]. The incidence, i.e., the reported cases of oral and oropharyngeal cancers, in the studies included in this review ranged from 34 to 154,525, with the latter utilizing multiple data sources such as the National Cancer Surveillance, epidemiology, and Surveillance, Epidemiology, and End Results (SEER) registries [14,16]. Within the studies reviewed, only nine included a comparison group, such as the urban population, while the rest collected data from the rural population alone. Most of the incidence reports consist of a significant rise in the percentage of the population affected by oral cancer in people above 40 years of age ranging from 45 to 88.6% of the people affected, compared to fewer being affected in the younger age group [14,40]. In the studies conducted in the USA, the range of percentage increase in incidence of oral cancer compared to the urban population was between 15.7% and 6.5% [20,47], while the highest increase due to rurality was reported in an Australian study affecting the Aboriginal rural population at 37.5% [42].

**Table 1:** Overview of studies included in the scoping review.

Citation	Participants	Data Analysis Sites	Methodology/Data Sources	Outcomes and Factors Identified
Benard et al. (2008) [20]	9464	Centers for Disease Control and Prevention National Program of Cancer Registries	Retrospective analysis Registers and county SES data and Surveillance, Epidemiology, and End Results (SEER) data from 1998 to 2003	Rurality—6.5% higher rates High school education—higher incidence in areas with <85% high school education Ethnicity—Hispanic and Asian/Pacific Islander race females showed lower ASR * Caucasians—lower rates in rural areas African Americans—higher rates than Caucasians in both rural and urban areas Income—lower income groups had higher incidence in males Poverty—higher poverty Current smoking—increased OSCC risk
Abreu et al. (2010) [40]	1197	The University of Western Australia	Retrospective analysis Western Australian Cancer Registry from 1982 to 2006	Rurality—11% higher rates in men in country areas Gender—men 2.4 times higher than women ASR * Age—88% in <40 years Indigenous status—non-indigenous 70% (men) and 55% (women) higher rates than indigenous
Frydrych et al. (2014) [42]	424	The University of Western Australia Curtin University	Retrospective analysis Western Australian Cancer Registry from 1990 to 1999	Rurality—higher incidence (68.8%) in rural Aboriginals Smoker—higher (44%) in Aboriginals Gender—higher (68.6%) in non-Aboriginal males Age at diagnosis—higher in 50–59 yrs (37.5%) Aboriginals Non-Aboriginal—higher in 60–69 yrs (30.2%)
Krupar et al. (2014) [27]	34	Department of Otolaryngology of the University Hospital Regensburg	Tissue analysis Cases from hospital records of OSCC patients diagnosed between 1993 and 2010	HPV prevalence: 50% Disease stage: advanced OSCC in 58.3% HPV positives
	85	Southern Germany Otolaryngology private practice OSCC	Tissue analysis	HPV prevalence: 16.1% Disease stage: advanced OSCC in 33.3% HPV positives
Walker et al. (2015) [23]	5473	University of British Columbia	Retrospective analysis British Columbia Cancer Registry from 1981 to 2009	Rurality: suburban cases increase 200%; rural—12% Gender: higher in males (64%)
Derbi et al. (2016) [41]	2801	The University of Western Australia	Retrospective analysis Western Australian Cancer Registry between 1982 and 2009	Tongue SCC Rurality: higher in rural (57%) ASR * increase—1.4 to 3.8 (1982 vs. 2009) Gender: males higher (69.2%) Age: highest ASR in 60–79 yrs (208.1)
Javadi et al. (2017) [17]	Reported on age-adjusted rates (per 100,000)	Southern Illinois University School of Medicine	Retrospective trend analysis Surveillance, Epidemiology, and End Results (SEER) 9 data from 1973 to 2012 and SEER-18 data from 2000 to 2012	Rurality: rural areas had sharpest increase in SCC trends Gender: male SCC rates higher than females in all rural areas Race: Whites significant decrease (1.85%) in trends
Delagranda et al. (2018) [35]	599	Public and private healthcare sectors	French data protection commission from 2009 to 2013	Gender: males higher (88.6%) Age: mean, 60 yrs (males), 62 yrs (females) Smoking: 89.6% (OPSCC) and 76.8% (OCSCC) Alcohol: 83.7% (OPSCC) and 71.6% (OCSCC) HPV infection: 32.4% (OPSCC) and 12.2% (OCSCC)
Radespiel-Tröger et al. (2018) [34]	18,947 (MPC)	Bavarian Health and Food Safety Authority	Retrospective analysis Bavarian cancer registry from 2003 to 2012	Rurality: higher (51.4%) cases Gender: males, overall (74.5%); rural (76%)
Pagedar et al. (2019) [14]	36,183 (OCC) 32,793 (OPC)	University of Iowa	Retrospective analysis National Cancer Institute Surveillance and Epidemiology (SEER) data from 1975 to 2015	Rurality: lower annual decline in incidence 0.5% vs. 2.6% (urban) for OCC; 4.6% increase vs. 2.6% (urban) for OPC Age: 45% in rural at 55–69 yrs Race: 99% White Stage: 47.9% localised SCC
Ghazawi et al. (2020) [33]	21,685 (OCC) 15,965 (OPC)	McGill University	Retrospective analysis Canadian Cancer Registry, Le Registre Quebecois du Cancer, Canadian Vital Statistics from 1992 to 2010	Gender: males higher 1.69 times (OCSCC) and 3.26 times (OPSCC) Age: highest incidence ≥90 yrs (OCSCC) and 60–69 yrs (OPSCC)
Harris et al. (2020) [47]	40,678	Harvard School of Dental Medicine	Retrospective analysis Surveillance, Epidemiology, and End Results (SEER) data from 1990 to 2015	Rural: increase in incidence 57.8% in rural vs. 42.1% in urban (2015) SCC grades: rural—higher grade 1 (well differentiated), urban—higher Grade 2 and 3 (moderately and poorly differentiated) Higher SCC rates in men: 70.4% Higher incidence: White (non-Hispanic) 96% Low income (<$50k): 37.9% higher OSCC Long term survival better in rural population SCC sites: rural—lower lip (22%); urban—base of tongue (24.9%)
Papenberg et al. (2020) [16]	154,525	West Virginia University	Retrospective analysis Data from NAACCR Epidemiology and SEER from 2007 to 2013	Sex: males higher (72.4%) Race: Whites higher (92%) SCC stage: stage IV (43%) HPV associated 61% Smoking: 20.7%
Clohessy et al. (2022) [37]	286	Calvary Mater Hospital	Retrospective analysis Data from digital medical records (DMR) from 2016 to 2017	Sex: males higher (80.4%) Age: <74 years higher (73.1%) Stage 4 disease 42% Patients lived 68.16 km from the multi-disciplinary team Cancer sites: cutaneous (35.3%) mucosa of the oral cavity including lips (29.4%) and pharynx (19.6%)
Cheng et al. (2022) [45]	92,685	West China Hospital of Stomatology	Retrospective analysis Data from Surveillance, Epidemiology, and End Results (SEER) from 1975 to 2018	Total annual percentage change (3.2) Age: >60 years (12.8) Sex: males higher (6.6%) Oral cancer (7.1%) Oropharyngeal cancer (3.9%) Black (15.2%)
Sun et al. (2023) [48]	9887	James Cook University	Queensland Cancer Registry: International Classification of Diseases 10th Revision from 1982 to 2018	Sites: moderately differentiated higher (49.45%), deaths higher (63%) Retromolar area higher (60.34%) Sex: male–female ratio 2.51–1 Oral SCC cases increased by 4.49-fold during study period
Ramadan et al. (2023) [46]	2000	National Cancer Institute	Surveillance, Epidemiology, and End Results (SEER) and 18 Census Track-level SES and Rurality Database from 2006 to 2018	Sites: oral tongue accounting for 44.6% Race: White people with tongue OCC 47%, Black population 36.8%, AAPI 49.2%, Hispanic 50.50% OCC highest in White Americans, 2.86 per 100,000 persons, and lowest in Black Americans 1.17 per 100,000
Liu et al. (2023) [49]	39,935	National Cancer Institute	Surveillance, Epidemiology, and End Results (SEER) and 18 Census Track-level SES and Rurality Database from 2000 to 2016	Race: NH White 54.8%, NH Black 36.1%, Pacific Islander 56.5% Sex: male—61.4% in NH Whites, NH Blacks 59.9% Age: mean age in NH Whites 66.03 and NH Blacks 62.64

* ASR: age-adjusted incidence rates (per 100,000); OPSCC: oropharyngeal squamous cell carcinoma; MPC: mouth and pharynx cancer; NH: non-Hispanic.

## 4. Discussion

This scoping review was conducted on studies involving oral cancer trends in rural and remote areas within the OECD member countries. All data collected in these studies included the sociodemographic profiles of rural populations, incidence and prevalence, and risk factors for oral and oropharyngeal cancers. Most of the studies included used retrospective analysis of data available from various state or national cancer registries.

It is evident that a substantially higher risk of oral cancer exists in the rural population in comparison to urban counterparts, reported to be ranging between 6.5% and 68.8% incidence in the rural population [20,42]. Generally, increasing age and long-standing tobacco and alcohol abuse are the leading causes of the increased incidence of oral cavity cancers in these regions. However, in most studies, the effect was more prominent in rural men than women. Harris et al. (2020) reported on 40,678 subjects in which they noted that a large percentage of rural men were affected by oral cancer compared to the urban population, but a similar number of women were affected in both rural and urban populations [47]. A study conducted in Queensland has shown the high prevalence of SCC in males over 60 years of age [48]. Similar trends were noted in another Australian study with 68.8% of rural men affected by oral cancer due to various risk behaviours such as tobacco and alcohol abuse along with HPV infections [42]. A study conducted in the USA presented the overall temporal trend of OC-OPC and the changes in fundamental factors, emphasising on the incidence and survival rate over the past 40 years [45]. Two studies conducted in the USA reported strong evidence to attribute rurality as the risk factor in oral cancer where a lack of awareness and the absence of prevention and early detection by healthcare providers are contributing to the soaring cases in recent decades [26,47].

The trends of oral and oropharyngeal cancers in the rural and remote areas of the OECD member countries included in this scoping review are directly related to the increasing age, gender, level of education and poverty, ethnicity, smoking status, and occupation. The advancement of age and lack of awareness about the lifestyle choices that are prevalent for a longer duration of time, along with other risk factors, such as regional isolation or remoteness, play a significant role in the increased incidence. A recent retrospective cohort study that examined the effect of remoteness on oral cancers noted that there was significant delay in the commencement of treatment from both the onset of symptoms (6 months vs. 3 months) and diagnosis (47 days vs. 36 days) in regional/remote patients, when compared to metropolitan patients in New South Wales, Australia [36]. This is particularly important since most patients undergo surgery in a metropolitan tertiary hospital through multiple specialist referrals between regional and metropolitan practices [36]. Additionally, the distance of patients’ residence from a multidisciplinary hospital has recently been reported to be a contributing factor, with the risk of being diagnosed with an advanced stage of cancer increasing approximately 1.5 times when the distance is over 100 km [37].

Poverty level, ethnicity, and education levels play a key role in the increasing incidence of oral cancer trends in the 21st century in the OECD countries’ rural populations. Poverty is directly associated with an increased incidence of oral cancer in countries like the USA, Canada, and Australia [20,47]. Specifically, 10–20% poverty status was associated with decreased oral and oropharyngeal cancer incidence rates [20]. Two other studies reported that the poverty level plays a role in both the increased incidence of oral cancer and the advancement of the disease [18,38]. Ethnicity is also known to be one of the significant socio-demographic factors that can affect the risk of oral cancers. Among the studies included, at least nine of the included studies reported that ethnicity was a major consideration for determining rising oral cancer cases among various ethnic groups [14,16,17,20,23,40,45,46,49]. Generally, Caucasians have a higher rate of incidence ranging from 70% in Australia to 99% in USA [14,40]. This may be partly due to the demographic variations within the study population wherein the proportion of Caucasians is significantly higher than non-Caucasians. Other ethnic groups with higher incidence include African Americans, Pacific Islanders, and Aboriginal people living in both rural and urban areas [20,41,42,48,49]. Another US study has reported that different races show significant variations in the incidence and prevalence rates of OCC and other forms of head and neck cancer [49].

The education level of the population is also known to affect the oral cancer incidence [26,28]. Specifically, higher incidence was noted in areas with under 85% of people with high school education, which can indirectly relate to lower awareness levels around the risk factors [20]. Tobacco and alcohol were identified as the main risk factors of oral cavity and oropharyngeal cancers in most countries including the USA, France, and Australia [16,20,35]. Two studies identified tobacco and alcohol as the main risk factors for oral cancer in all age groups, with particularly increased incidence of SCC in the age group between 50 and 59 years [33,42].

Various occupational factors have been associated with an increased risk of head and neck cancers, including oral cancers. In a case–control study, long term unemployment was reported to increase the risk of oral cancers by 2.9 times in one study [50,51]. Notably, the unemployed patients with oral cancer had significantly higher smoking and alcohol consumption rates, which may in part explain the higher incidence [50]. Unemployment has also been correlated with cancer mortality at both the individual and community level [52]. A large INHANCE (International Head and Neck Cancer Epidemiology) Consortium-led analysis of pooled case–control studies from Western Europe, Latin America, Germany, and France analysed the role of occupational socioeconomic risks for head and neck cancer comprehensively [53]. This study reported that occupational socioeconomic prestige, position, and manual work increased the risk of head and neck cancer, especially if employed in the industry for 10 years or over, after adjusting for smoking and alcohol use [53]. A range of professions that had an increased risk (odds) of oral and oropharyngeal cancer were identified, including loggers, dairy farmers, and manual labourers in building and construction industries like bricklayers, painters, roof workers, reinforced concreters, road (asphalt) workers, drivers (lorries, vans, and earthmovers), and cargo handlers, particularly in males, mostly due to the male-dominated industries [54,55,56]. Exposure to harmful chemicals and known carcinogens including cement dust, asbestos, polycyclic aromatic hydrocarbons, inorganic dusts, and solvents over a long period of time were identified as the potential reasons for this increased risk [57,58,59]. Additionally, two US studies included in this review also reported that occupations with low income and living in rural areas were associated with an increased risk of oral cancers [20,47].

Human papillomavirus (HPV) is increasingly recognized as a known risk for some forms of head and neck cancers, particularly oral cancers, with rates of cancers with HPV on the rise while non-HPV cancers are on a decline [29]. Traditionally, two types of clinicopathological forms of oral and oropharyngeal cancers are described in the literature, based on the presence or absence of HPV [60]. HPV-positive tumours are known to clinically present with early lymph node metastasis, albeit better responsiveness to radiation and chemotherapy has been reported with up to a 58% reduction in the risk of death [61,62]. Furthermore, a significant correlation between the sexual practices and the awareness as well as vaccination status for HPV-related oropharyngeal cancer incidences has been identified [63]. An estimated 60–70% of oropharyngeal cancers are associated with HPV infection in developed countries such as the United States, compared to under 10% in developing or underdeveloped regions [2,64,65,66]. At least three of the studies included in our review identified an association between HPV infection and oropharyngeal squamous cell carcinoma, with increased risk ranging from 12 to 61% [16,27,35]. Additionally, higher education levels have also been associated with increased HPV-associated oral cancers.

## 5. Conclusions

This scoping review revealed the negative influence of rurality in oral cancer trends, particularly among socioeconomically deprived, aged, and geographically distant communities. This scoping review confirmed the limited knowledge and variations in the literature around the rural remote population in many OECD member countries vulnerable to cancer, where a lack of primary healthcare centres and tertiary cancer care facilities are expected. The gaps evident in the data around incidence, prevalence, and risk factors in many other OECD countries support the need for further population-based studies in the region, specifically in regional rural and remote areas, and a comparison to the urban population is essential. Furthermore, identifying the at-risk population can assist in designing awareness programs to implement effective intervention strategies targeting specific communities.

## Supplementary Materials

The following supporting information can be downloaded at: https://www.mdpi.com/article/10.3390/cancers16172957/s1, Table S1: Search strategy used for retrieval of records.
