# Supplementary material for: Influence of Rurality on Oral Cancer Trends among Organisation for Economic Co-Operation and Development (OECD) Member Countries—A Scoping Review
Source: Cancers (Basel). 2024 Aug 24;16(17):2957. doi: 10.3390/cancers16172957 (PMC11394544; doi:10.3390/cancers16172957)
Supplement: Supplementary file 1 [file cancers-16-02957-s001.zip › cancers-3108915-supplementary.pdf]

Supplementary Table S1. Search strategy used for retrieval of records.

Database	Search String
Medline (Ovid)	incidence/ or prevalence/ or mortality/<br>(rural or remote).mp. [mp=title, abstract, original title, name of substance word, subject heading word, floating sub-heading word, keyword heading word, organism supplementary concept word, protocol supplementary concept word, rare disease supplementary concept word, unique identifier, synonyms]<br>("oral cancer" or "mouth neoplasms").mp. [mp=title, abstract, original title, name of substance word, subject heading word, floating sub-heading word, keyword heading word, organism supplementary concept word, protocol supplementary concept word, rare disease supplementary concept word, unique identifier, synonyms]
PubMed	((((oral cancer) AND (incidence)) AND (prevalence)) AND (risk factors)) AND (rural OR remote)<br>("mouth neoplasms"[MeSH Terms] OR ("mouth"[All Fields] AND "neoplasms"[All Fields]) OR "mouth neoplasms"[All Fields] OR ("oral"[All Fields] AND "cancer"[All Fields]) OR "oral cancer"[All Fields]) AND ("epidemiology"[MeSH Subheading] OR "epidemiology"[All Fields] OR "incidence"[All Fields] OR "incidence"[MeSH Terms] OR "incidences"[All Fields] OR "incident"[All Fields] OR "incidents"[All Fields]) AND ("epidemiology"[MeSH Subheading] OR "epidemiology"[All Fields] OR "prevalence"[All Fields] OR "prevalence"[MeSH Terms] OR "prevalance"[All Fields] OR "prevalences"[All Fields] OR "prevalence s"[All Fields] OR "prevalent"[All Fields] OR "prevalently"[All Fields] OR "prevalents"[All Fields]) AND ("risk factors"[MeSH Terms] OR ("risk"[All Fields] AND "factors"[All Fields]) OR "risk factors"[All Fields]) AND ("rural"[All Fields] OR "ruralities"[All Fields] OR "rurality"[All Fields] OR "rurally"[All Fields] OR "ruralness"[All Fields] OR "rurals"[All Fields] OR ("remote"[All Fields] OR "remotely"[All Fields] OR "remoteness"[All Fields] OR "remotes"[All Fields]))
Scopus	("oral cancer" OR "mouth neoplasms" AND "incidence" AND "prevalence" AND "risk factors" AND "patterns" AND "rural" OR "remote")
CINAHL	((MH "Prevalence") AND (MH "Incidence") AND "oral cancer AND (incidence AND prevalence AND risk factors) AND (rural OR remote)") AND oral cancer
